# Epidemiological and clinical characteristics of macrolide-resistant *Bordetella pertussis* in southern China: an 8-year retrospective study

**DOI:** 10.1186/s12879-026-12911-9

**Published:** 2026-03-03

**Authors:** Zengrui Lin, Hongmei Wang, Cuijuan Liao, Guangcheng Deng, Jinjun Zheng, Ying Zhang, Xinxin Liu, Yuchun Long, Shufeng Tian, Biao Sun, Kun Tan, Jikui Deng

**Affiliations:** Department of Infectious Diseases, Shenzhen Children’s Hospital, No. 7019 Yitian Road, Futian District, Shenzhen, Guangdong 518038 China

**Keywords:** *Bordetella pertussis*, Macrolide resistance, Minimum inhibitory concentration, COVID-19 pandemic

## Abstract

**Background:**

Recently, macrolide-resistant *Bordetella pertussis* (MRBP) had emerged in China. The aim of this study was to evaluate the prevalence and clinical characteristics of MRBP in Shenzhen, southern China.

**Methods:**

We enrolled patients with culture-confirmed pertussis at Shenzhen Children’s Hospital from January 2016 to December 2023. Demographic and clinical data of the patients were collected. The antimicrobial susceptibility of *Bordetella pertussis* isolates was evaluated, and the minimum inhibitory concentrations (MICs) against erythromycin, trimethoprim-sulfamethoxazole (TMP-SMZ), levofloxacin, ampicillin, and ceftriaxone were determined using the E-test method. Patients were categorized into macrolide-resistant (MR) and macrolide-sensitive (MS) groups according to the erythromycin susceptibility of their isolates. Group comparisons were performed using the Mann-Whitney U test or chi-square test, as appropriate. Multivariable logistic regression was performed to identify factors independently associated with MRBP infection. A two-sided *p* < 0.05 was considered statistically significant.

**Results:**

A total of 678 patients were enrolled. The MIC_90_ of erythromycin, TMP-SMZ, levofloxacin, ampicillin, and ceftriaxone was > 256 mg/L, 0.38 mg/L, 0.5 mg/L, 1 mg/L, 0.25 mg/L, respectively. The proportion of MRBP strains significantly increased after early 2020 (44.5% in 2016–2019 vs. 89.1% in 2020–2023, *p* < 0.001). Compared with the MS group, the MR group was older and had higher diphtheria-tetanus-acellular pertussis (DTaP) vaccination rate, longer hospital stay, longer antibiotic use before culture and during hospitalization, more TMP-SMZ use, and more persistently positive cultures (all *p* < 0.001). Binary logistic regression including age, study period, and DTaP vaccination status showed that only the study period (2020–2023 vs. 2016–2019) was independently associated with MRBP infection (OR = 9.516, 95% CI 5.738–15.781, *p* < 0.001).

**Conclusions:**

The prevalence of MRBP increased markedly in southern China after the COVID-19 pandemic. TMP-SMZ, levofloxacin, ampicillin, and ceftriaxone remained effective against *B. pertussis* in vitro. MRBP infection was associated with greater antibiotic exposure, longer hospitalization, TMP-SMZ use, and persistently positive cultures.

## Background

Pertussis (whooping cough), caused by the Gram-negative bacterium *Bordetella pertussis* (*B. pertussis*), is a highly contagious respiratory disease [1–3]. Clinical manifestations in adults and adolescents are usually mild or atypical. Transmission of pertussis commonly occurs in households and communities [2, 4]. Unvaccinated neonates and infants are at higher risk of infection and developing severe complications [2, 5]. The introduction and widespread use of pertussis vaccine have significantly reduced the incidence of pertussis [3, 6]. Nevertheless, since the 1980s, there has been a global resurgence of pertussis, even in industrialized countries with high vaccination coverage rates [7, 8].

Antibiotics are used to eradicate *B. pertussis* from the nasopharynx of infected individuals and thereby prevent further transmission [1, 9]. Early-stage antimicrobial therapy can alleviate symptoms and shorten the disease course [9]. There are relatively few evidence-based antimicrobials for pertussis treatment and chemoprophylaxis. Macrolide is recommended as the first-line agent in children due to its proven clinical effectiveness and safety [1, 9, 10]. Alternative therapeutic choices may include trimethoprim-sulfamethoxazole (TMP-SMZ), β-lactams, clindamycin, and fluoroquinolones [9]. However, over the past decade, circulating *B. pertussis* strains in China have exhibited high levels of resistance to macrolides, raising public health concerns [11].

Shenzhen, a highly connected metropolis in southern China with a population of nearly 20 million, may provide favorable conditions for rapid transmission once macrolide-resistant *Bordetella pertussis* (MRBP) is established. This study aimed to characterize the local antimicrobial susceptibility profiles of *B. pertussis* isolates, and to describe the associated clinical characteristics.

## Methods

### Study population

Patients diagnosed with pertussis based on positive culture for *B. pertussis* at Shenzhen Children’s Hospital between January 2016 and December 2023 were enrolled. Cases were excluded if the corresponding isolate was unavailable for antimicrobial susceptibility testing or if their medical records lacked key information. Demographic and clinical characteristics, including age, sex, diphtheria-tetanus-acellular pertussis (DTaP) vaccination status, and hospitalization status (outpatient or inpatient), were obtained from the electronic medical records. For hospitalized patients, additional data, including length of hospital stay, intensive care unit (ICU) admission, readmission, and antibiotic prescriptions, were also collected.

### Bacterial culture

The patients’ nasopharyngeal specimens were collected and then incubated on charcoal agar plate (OXOID, UK) supplemented with 10% defibrinated sheep blood and cephalexin at 37 degrees Celsius for up to 7 days. Bacteria colonies that were round, smooth, convex, silver-gray colored, and rod-shaped were identified as *Bordetella* species. Suspected colonies were further confirmed through the agglutination test.

### Antimicrobial susceptibility testing

The E-test method was used to determine the antimicrobial susceptibility of *B. pertussis* against erythromycin, ampicillin, ceftriaxone, levofloxacin, and TMP-SMZ. E-test strips (Liofilchem, Italy) were applied onto the agar plate. Minimum inhibitory concentration (MIC) values were read after 72 h, in accordance with the manufacturer’s instructions. The drug concentrations of the strips tested were as follows: erythromycin, 0.016 to 256 mg/L; ampicillin, 0.016 to 256 mg/L; ceftriaxone, 0.002 to 32 mg/L; levofloxacin, 0.002 to 32 mg/L; and TMP-SMZ, 0.002 to 32 mg/L. *Haemophilus influenzae* ATCC49247 and *Staphylococcus aureus* ATCC29213 were used for quality control.

### Statistical analysis

Data were analyzed using WHONET (version 5.6) and SPSS (version 27.0). Patients were categorized into macrolide-resistant (MR) and macrolide-sensitive (MS) groups according to erythromycin susceptibility results of their isolates. Normally distributed numerical data were presented as mean ± standard deviation ($\:\overline{X}$ ± S). Non-normally distributed data were presented as median (interquartile range) (M [IQR]). Between-group comparisons were conducted using Student’s t-test or the Mann-Whitney U test for continuous variables, and the chi-square test or Fisher’s exact test for categorical variables, as appropriate. Binary logistic regression was used to identify factors independently associated with macrolide resistance, with covariates selected based on clinical relevance and informed by univariable analyses. A two-sided *p*-value of < 0.05 was considered statistically significant.

## Results

### Demographic and clinical characteristics

Over the eight-year study period (January 2016 to December 2023), a total of 678 culture-confirmed pertussis cases were identified. Repeated culture testing was performed in 244 (36.0%) patients, 155 (63.5%) of whom yielded *B. pertussis* isolates on more than one occasion (persistently culture-positive). The cohort consisted of 382 (56.3%) males and 296 (43.7%) females, with a median age of 4 (2, 10) months. Immunization history at first diagnosis was unavailable for 69 patients. Among those with available immunization history records (*n* = 609), DTaP vaccination status of the patients was as follows: unvaccinated, 338 (55.5%); one dose, 113 (18.6%); two doses, 32 (5.2%); three doses, 36 (5.9%); and four doses, 90 (14.8%). A total of 556 (82.0%) patients were assessed by clinicians as requiring hospitalization, and 19 of whom were discharged of their own volition. We analyzed the antibiotic prescriptions for the remaining 537 inpatients. Among them, 364 (67.8%) had received antibiotic treatment prior to the first nasopharyngeal sample collection. The number of patients receiving macrolide, TMP-SMZ, and β-lactam up to the time of discharge was 531 (98.9%), 143 (26.6%), and 314 (58.5%), respectively. Sixteen (3.0%) patients were admitted to ICU, 55 (10.2%) patients were readmitted, and no deaths were reported. 

### Antimicrobial susceptibility testing of *B. pertussis*

**Fig. 1:**
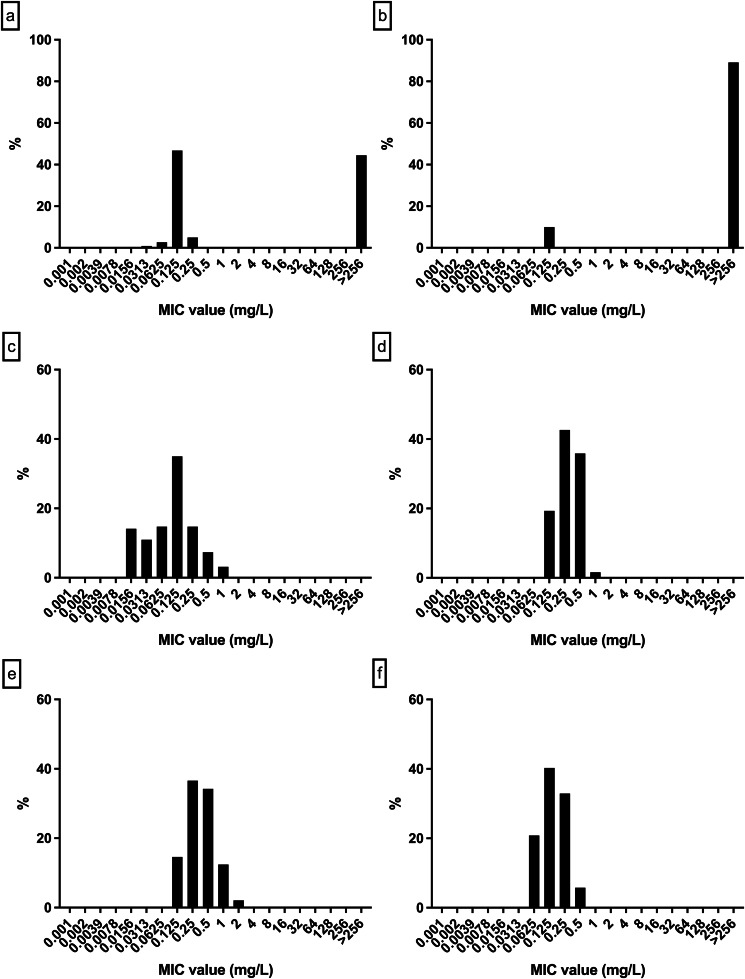
MIC distributions in erythromycin, trimethoprim-sulfamethoxazole, levofloxacin, ampicillin, and ceftriaxone for *B. pertussis* strains; **a**. erythromycin (2016–2019); **b**. erythromycin (2020–2023); **c**. trimethoprim-sulfamethoxazole (TMP-SMZ); **d**. levofloxacin; **e**. ampicillin; **f**. ceftriaxone

**Fig. 2:**
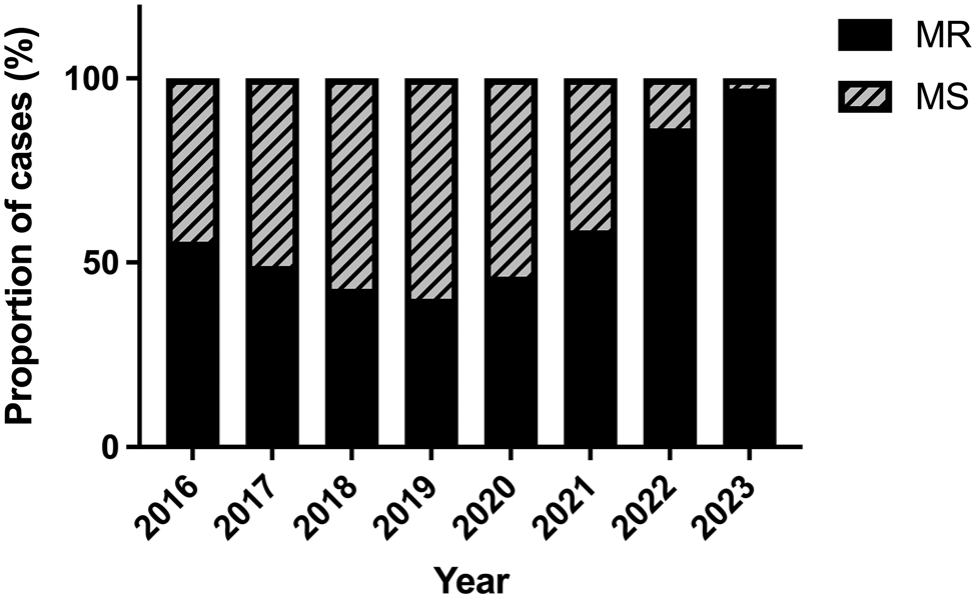
Proportions of macrolide-resistant (MR) and macrolide-sensitive (MS) *B. pertussis*, 2016–2023

For patients with persistently positive cultures, only the first isolate per patient was retained for antimicrobial susceptibility testing, and subsequent isolates were excluded. Antimicrobial susceptibility testing for TMP-SMZ, levofloxacin, ampicillin, and ceftriaxone was performed for 678 *B. pertussis* isolates, whereas erythromycin susceptibility was assessed for 666 isolates due to limited reagent availability. Table 1 presents the MIC range, MIC_50_, and MIC_90_ for erythromycin, TMP-SMZ, levofloxacin, ampicillin, and ceftriaxone across the two study periods (2016–2019 and 2020–2023). The MIC_50_ of erythromycin, TMP-SMZ, levofloxacin, ampicillin, and ceftriaxone was > 256 mg/L, 0.064 mg/L, 0.25 mg/L, 0.25 mg/L, 0.125 mg/L, respectively. The MIC_90_ of erythromycin, TMP-SMZ, levofloxacin, ampicillin, and ceftriaxone was > 256 mg/L, 0.38 mg/L, 0.5 mg/L, 1 mg/L, 0.25 mg/L, respectively. Figure 1 demonstrates the MIC distributions. Currently, neither the Clinical and Laboratory Standards Institute (CLSI) nor the European Committee on Antimicrobial Susceptibility Testing (EUCAST) has standardized the MIC cut-offs for *B. pertussis*. A total of 395 (59.3%) strains with MIC > 256 mg/L were undoubtedly resistant to erythromycin, while one strain with MIC of 4 mg/L and 270 strains (40.7%) with low MIC values (ranging from 0.016 to 0.25 mg/L) were considered sensitive. The proportion of MRBP strains from 2016 to 2023 was 25/45 (55.6%), 47/96 (49.0%), 63/147 (42.9%), 63/157 (40.1%), 6/13 (46.1%), 10/17 (58.8%), 38/44 (86.4%), and 143/147 (97.3%), respectively (see Fig. 2). The proportion of MRBP strains significantly increased after early 2020, from 198/445 (44.5%) in 2016–2019 to 197/221 (89.1%) in 2020–2023 (χ2 = 121.962, *p* < 0.001).

**Table 1:** Antimicrobial susceptibility of erythromycin, TMP-SMZ, levofloxacin, ampicillin, and ceftriaxone for *B. pertussis*

Year	Antibiotics	No. of strains	MIC range (mg/L)	MIC_50_ (mg/L)	MIC_90_ (mg/L)
2016–2019	Erythromycin	445	0.016- > 256	0.125	> 256
	TMP-SMZ	445	0.008-1	0.064	0.25
	Levofloxacin	457	0.032-1.5	0.25	0.5
	Ampicillin	457	0.064-2	0.25	1
	Ceftriaxone	457	0.047-1	0.125	0.25
2020–2023	Erythromycin	221	0.016- > 256	> 256	> 256
	TMP-SMZ	221	0.008-1	0.094	0.5
	Levofloxacin	221	0.008-1	0.25	0.5
	Ampicillin	221	0.125-16	0.5	1
	Ceftriaxone	221	0.064-0.5	0.25	0.25
Total	Erythromycin	666	0.016- > 256	> 256	> 256
	TMP-SMZ	666	0.008-1	0.064	0.38
	Levofloxacin	678	0.008-1.5	0.25	0.5
	Ampicillin	678	0.064-16	0.25	1
	Ceftriaxone	678	0.047-1	0.125	0.25

### Macrolide resistance and patient characteristics

As summarized in Table 2, univariate analysis showed that MRBP was more frequently identified in older patients (age, 4.5 [3, 12] vs. 4 [2, 6] months, *p* < 0.001). The DTaP vaccination rate was also higher in the MR group than in the MS group (181/360 [50.3%] vs. 86/237 [36.3%], *p* < 0.001). Among hospitalized patients, the MR group had longer length of hospital stay (9 [7, 13] vs. 8 [6, 11] days, *p* < 0.001) and longer antibiotic use both before culture (6 [4, 10] vs. 3 [1, 5] days, *p* < 0.001) and during hospitalization (8 [6, 11] vs. 7 [5, 9] days, *p* < 0.001), comparing with MS group. TMP-SMZ was prescribed more frequently in the MR group than in the MS group (139/310 [44.8%] vs. 4/215 [1.9%], *p* < 0.001). Among the 244 patients who underwent repeated culture testing, persistently culture-positive results were more common in the MR group than in the MS group (132/173 [76.3%] vs. 23/71 [32.4%], *p* < 0.001). No significant differences were observed between the two groups regarding sex, hospitalization status, ICU admission, readmission, or macrolide/β-lactam administration (all *p* > 0.05). In the binary logistic regression (Table 3), we included two study periods (2020–2023 vs. 2016–2019 baseline), age, and DTaP vaccination status as covariates. Only the study period (2020–2023) remained independently associated with MRBP infection (OR = 9.516, 95%CI 5.738–15.781, *p* < 0.001). Age (OR = 1.001, 95%CI 0.992–1.011, *p* = 0.767) and DTaP vaccination status (OR = 1.487, 95%CI 0.989–2.234, *p* = 0.056) were not statistically significant after adjustment.

**Table 2:** Demographic and clinical characteristics of patients with MRBP and MSBP infection (Univariable comparisons)

		MR (n=395)	MS (n=271)	χ2 or Z	*p*-value
Age (months)	Median (IQR)	4.5 (3, 12)	4 (2, 6)	5.236	< 0.001
Sex, male	N (%)	227 (57.5)	148 (54.6)	0.533	0.465
DTaP vaccination status^1^	N (%)	181 (50.3)	86 (36.3)	11.316	< 0.001
Inpatient^2^	N (%)	310 (80.7)	215 (81.7)	0.106	0.745
Length of hospital stay (days)^3^	Median (IQR)	9 (7, 13)	8 (6, 11)	4.589	< 0.001
ICU admission^3^	N (%)	10 (3.2)	6 (2.8)	0.081	0.775
Re-admission^3^	N (%)	29 (9.4)	25 (11.6)	0.711	0.399
Length of antibiotic use before culture (days)^3^	Median (IQR)	6 (4, 10)	3 (1, 5)	8.419	< 0.001
Length of antibiotic use during hospitalization (days)^3^	Median (IQR)	8 (6, 11)	7 (5, 9)	5.461	< 0.001
Macrolide administration^3^	N (%)	308 (99.4)	211 (98.1)	1.660	0.198
β-lactam administration^3^	N (%)	188 (60.6)	118 (54.9)	1.733	0.188
TMP-SMZ administration^3^	N (%)	139 (44.8)	4 (1.9)	118.320	< 0.001
Persistently positive cultures^4^	N (%)	132 (76.3)	23 (32.4)	41.882	< 0.001

1. DTaP vaccination status was unknown for 69 patients

2. Excluding 19 patients who were discharged of their own volition

3. Data were available for 525 inpatient cases (215 with MSBP and 310 with MRBP)

4. For 244 patients who underwent repeated culture testing

**Table 3:** Demographic and clinical characteristics of patients with MRBP and MSBP infection (Binary logistic regression)

		MR (n=395)	MS (n=271)	*p*-value	Odds Ratio (95%CI)
Age (months)	Median (IQR)	4.5 (3, 12)	4 (2, 6)	0.767	1.001 (0.992–1.011)
Study period, 2020–2023	N (%)	197 (49.9)	24 (8.9)	< 0.001	9.516 (5.738–15.781)
DTaP vaccination status^1^	N (%)	181 (50.3)	86 (36.3)	0.056	1.487 (0.989–2.234)

1. DTaP vaccination status was unknown for 69 patients

## Discussion

The epidemiology of MRBP is highly heterogeneous worldwide. Sustained high-prevalence MRBP circulation has been reported predominantly in mainland China, whereas MRBP has historically been sporadic in most other regions [7]. In our study, the prevalence of MRBP was high, and a notable increase was observed after early 2020, consistent with findings from Shanghai, where the proportion of MRBP increased from 36.4% in 2016 to 97.2% in 2022 [12]. Several epidemiological factors may have contributed to the emergence and expansion of MRBP. During the coronavirus disease 2019 (COVID-19) pandemic, non-pharmaceutical interventions (e.g., mask-wearing, social distancing, and public restrictions) were widely implemented [13]. Similar to other respiratory infectious diseases, the incidence of pertussis declined sharply during this period. Following the relaxation of these measures, pertussis rebounded in the post-COVID-19 pandemic period, potentially facilitating the rapid dissemination and the selection of successful lineages. During the post-COVID-19 pandemic resurgence of pertussis in Europe, Finland reported its first MRBP isolate in 2024 despite long-term surveillance indicating sustained macrolide susceptibility, and France also reported macrolide resistance in the same year [14, 15]. Other Asian countries including Iran, Japan, and Cambodia have reported MRBP only occasionally, and available genomic evidence suggests that the detected Japanese MRBP strains are phylogenetically related to Chinese lineages [16–19].

In the context of increasing antimicrobial resistance, a major clinical concern is whether MRBP contributes to severe pertussis or poor outcomes. Although MRBP remains uncommon outside mainland China and severe pertussis is relatively rare, fatal MRBP case was still reported in Japan in 2024 [18, 19]. Previous studies uncovered the clinical characteristics and prognostic factors associated with MRBP infection. In our cohort, macrolide resistance was not associated with clinical severity, as reflected by comparable hospitalization and ICU admission rates between the MR and MS groups, suggesting that macrolide resistance alone may not be a primary determinant of severity in this setting.

In our study, individuals harboring resistant strains experienced longer durations of antibiotic therapy and more frequent persistently positive cultures, suggesting a suboptimal response to empiric antibiotics. Prolonged treatment courses may also increase opportunities for onward transmission. Despite the emergence of macrolide resistance, erythromycin and azithromycin are still recommended as first-line therapeutic agents in most guidelines [9, 20]. Throughout our eight-year surveillance, the MIC values detected for TMP-SMZ, levofloxacin, ceftriaxone, and ampicillin were within acceptable ranges, indicating their potent antimicrobial activity against *B. pertussis* in vitro. In our clinical practice, a subset of patients were treated with second-line therapy using TMP-SMZ, an alternative antibiotic for patients over two months of age without G6PD deficiency, based on susceptibility testing reports and/or clinicians’ assessment of treatment response. To our knowledge, TMP-SMZ-resistant *B. pertussis* isolates have not been reported. However, both the TMP-SMZ MIC_50_ (0.064 mg/L vs. 0.094 mg/L) and MIC_90_ (0.25 mg/L vs. 0.5 mg/L) were higher in the post-COVID-19 pandemic period than in the pre-COVID-19 pandemic period. A recent study in northern China also reported the increase of maximum MIC of TMP-SMZ [21]. β-lactam is the most commonly used antibiotic in pediatric practice. Previous studies demonstrated low level of MIC of β-lactams in *B. pertussis* isolates, and reported that the β-lactam/β-lactamase inhibitor combinations cefoperazone-sulbactam and piperacillin-tazobactam can alleviate clinical symptoms in pertussis cases and achieve higher clearance rates of MRBP [22, 23]. However, both agents require intravenous administration. Most in vitro studies have shown that *B. pertussis* isolates were sensitive to fluoroquinolone [24–27]. However, six *B. pertussis* strains were reported as resistant to nalidixic acid (a quinolone derivative) [28]. The clinical use of systemic fluoroquinolone in children is limited due to safety considerations. Given the discrepancy between current guidelines and the increasing prevalence of MRBP, further studies are warranted to evaluate the clinical effectiveness and safety of alternative antimicrobial regimens.

Our study has several limitations. First, case inclusion was solely based on cultures. Therefore, pertussis patients diagnosed by polymerase chain reaction (PCR) or serology were not included. Second, bacterial clearance before specimen collection may have resulted in selection bias, especially for macrolide-sensitive *B. pertussis* infections. Third, co-infections with other pathogens may have influenced the antibiotic prescriptions. Finally, the assessment of clinical severity and outcomes was limited to hospitalization status, ICU admission and mortality, while more parameters (e.g., dyspnea, pneumonia, pulmonary hypertension, pertussis encephalopathy, and need for mechanical ventilation) were not assessed.

## Conclusions

The prevalence of circulating MRBP strains in Shenzhen, southern China, increased markedly after the COVID-19 pandemic. TMP-SMZ, levofloxacin, ampicillin, and ceftriaxone remained effective against *B. pertussis* in vitro. MRBP infection was associated with greater antibiotic exposure, longer hospitalization, TMP-SMZ use, and persistently positive cultures. Further studies are needed to evaluate the clinical efficacy of current treatment protocols for pertussis and to conduct genomic epidemiological studies on antimicrobial resistance.

## Data Availability

The datasets used and/or analysed during the current study are available from the corresponding author on reasonable request.

## Abbreviations

MRBP: Macrolide-resistant *Bordetella pertussis*
*B. pertussis*: *Bordetella pertussis*
MIC: Minimum inhibitory concentration
TMP-SMZ: Trimethoprim-sulfamethoxazole
COVID-19: Coronavirus disease 2019
DTaP: Diphtheria-tetanus-acellular pertussis
ICU: Intensive care unit

## References

[1] Kilgore PE, Salim AM, Zervos MJ, Schmitt HJ. Pertussis: Microbiology, Disease, Treatment, and Prevention. Clin Microbiol Rev. 2016;29(3):449–86. 27029594 10.1128/CMR.00083-15 PMC4861987

[2] Guiso N. Bordetella pertussis: why is it still circulating? J Infect. 2014;68(Suppl 1):S119–24. 24103807 10.1016/j.jinf.2013.09.022

[3] Kerr JR, Matthews RC. Bordetella pertussis infection: pathogenesis, diagnosis, management, and the role of protective immunity. Eur J Clin Microbiol Infect Dis. 2000;19(2):77–88. 10746492 10.1007/s100960050435

[4] Craig R, Kunkel E, Crowcroft NS, et al. Asymptomatic Infection and Transmission of Pertussis in Households: A Systematic Review. Clin Infect Dis. 2020;70(1):152–61. 31257450 10.1093/cid/ciz531

[5] Jordan I, Felipe A, Balaguer M, et al. Morbidity and mortality risk factors of pertussis in pediatrics. J Infect. 2017;74(1):97–100. 27646512 10.1016/j.jinf.2016.09.002

[6] Zepp F, Heininger U, Mertsola J, et al. Rationale for pertussis booster vaccination throughout life in Europe. Lancet Infect Dis. 2011;11(7):557–70. 21600850 10.1016/S1473-3099(11)70007-X

[7] Ivaska L, Barkoff AM, Mertsola J, He Q. Macrolide Resistance in Bordetella pertussis: Current Situation and Future Challenges. Antibiot (Basel). 2022;11(11):1570. 10.3390/antibiotics11111570 PMC9686491 36358225

[8] Locht C. Live pertussis vaccines: will they protect against carriage and spread of pertussis? Clin Microbiol Infect. 2016;22(Suppl 5):S96–102. 28341014 10.1016/j.cmi.2016.05.029

[9] Tiwari T, Murphy TV, Moran J. Recommended antimicrobial agents for the treatment and postexposure prophylaxis of pertussis: 2005 CDC Guidelines. MMWR Recomm Rep. 2005;54(RR–14):1–16. 16340941

[10] Subspecialty Group of Infectious Diseases, the Society of Pediatrics, Chinese Medical Association. Editorial Board, Chinese Journal of Pediatrics. Recommendation for diagnosis and treatment of Chinese children with pertussis. Zhonghua Er Ke Za Zhi. 2017;55(8):568–72. 28822429 10.3760/cma.j.issn.0578-1310.2017.08.004

[11] Feng Y, Chiu CH, Heininger U, Hozbor DF, Tan TQ, König CW. Emerging Macrolide Resistance in Bordetella Pertussis in Mainland China: Findings and warning from the global pertussis initiative. Lancet Reg Health West Pac. 2021;8:100098. 34327426 10.1016/j.lanwpc.2021.100098 PMC8315362

[12] Fu P, Zhou J, Yang C, et al. Molecular evolution and increasing macrolide resistance of Bordetella pertussis, Shanghai, China, 2016–2022. Emerg Infect Dis. 2024;30(1):29–38. 38146984 10.3201/eid3001.221588 PMC10756392

[13] Geng Y, Zhang L. Impact of non-pharmaceutical interventions during COVID-19 pandemic on pertussis, scarlet fever and hand-foot-mouth disease in China. J Infect. 2022;84(2):e13–5. 34953908 10.1016/j.jinf.2021.12.023 PMC8694816

[14] Miettinen M, Barkoff AM, Nyqvist A, et al. Macrolide-resistant Bordetella pertussis strain identified during an ongoing epidemic, Finland, January to October 2024. Euro Surveill. 2024;29(49):2400765. 39639816 10.2807/1560-7917.ES.2024.29.49.2400765 PMC11650481

[15] Rodrigues C, Bouchez V, Soares A, et al. Resurgence of Bordetella pertussis, including one macrolide-resistant isolate, France, 2024. Euro Surveill. 2024;29(31):2400459. 39092529 10.2807/1560-7917.ES.2024.29.31.2400459 PMC11295439

[16] Koide K, Yao S, Chiang CS, et al. Genotyping and macrolide-resistant mutation of Bordetella pertussis in East and South-East Asia. J Glob Antimicrob Resist. 2022;31:263–9. 36270447 10.1016/j.jgar.2022.10.007 PMC9750937

[17] Mirzaei B, Bameri Z, Babaei R, Shahcheraghi F. Isolation of High Level Macrolide Resistant Bordetella pertussis Without Transition Mutation at Domain V in Iran. Jundishapur J Microbiol. 2015;8(7):e18190. 26396713 10.5812/jjm.8(5)2015.18190 PMC4575774

[18] Koide K, Uchitani Y, Yamaguchi T, et al. Whole-genome comparison of two same-genotype macrolide-resistant Bordetella pertussis isolates collected in Japan. PLoS ONE. 2024;19(2):e0298147. 38359004 10.1371/journal.pone.0298147 PMC10868825

[19] Iwasaki T, Koide K, Kido T, et al. Fatal case of macrolide-resistant Bordetella pertussis infection, Japan, 2024. J Infect Chemother. 2025;31(7):102727. 40348379 10.1016/j.jiac.2025.102727

[20] Pediatric Infection Group, Chinese Society of Infectious Diseases, Infection Group, Pediatric Expert Committee of National Health Commission Capacity Building and Continuing Education, National Medical Center for Infectious Diseases. Guidelines for diagnosis and management and prevention of pertussis of China (2024 edition). Zhonghua Yi Xue Za Zhi. 2024;104(15):1258–79. 38637166 10.3760/cma.j.cn112137-20240124-00179

[21] Hu Y, Zhou L, Du Q, et al. Sharp rise in high-virulence Bordetella pertussis with macrolides resistance in Northern China. Emerg Microbes Infect. 2025;14(1):2475841. 40042368 10.1080/22221751.2025.2475841 PMC11921162

[22] Hua CZ, Wang HJ, Zhang Z, et al. In vitro activity and clinical efficacy of macrolides, cefoperazone-sulbactam and piperacillin/piperacillin-tazobactam against Bordetella pertussis and the clinical manifestations in pertussis patients due to these isolates: A single-centre study in Zhejiang Province, China. J Glob Antimicrob Resist. 2019;18:47–51. 30710647 10.1016/j.jgar.2019.01.029

[23] Mi YM, Hua CZ, Fang C, et al. Effect of Macrolides and β-lactams on Clearance of Bordetella pertussis in the Nasopharynx in Children With Whooping Cough. Pediatr Infect Dis J. 2021;40(2):87–90. 33021592 10.1097/INF.0000000000002911

[24] Yang Y, Yao K, Ma X, Shi W, Yuan L, Yang Y. Variation in Bordetella pertussis Susceptibility to Erythromycin and Virulence-Related Genotype Changes in China (1970–2014). PLoS ONE. 2015;10(9):e0138941. 26406905 10.1371/journal.pone.0138941 PMC4583996

[25] Jakubů V, Zavadilová J, Fabiánová K, Urbášková P. Trends in the Minimum Inhibitory Concentrations of Erythromycin, Clarithromycin, Azithromycin, Ciprofloxacin, and Trimethoprim/Sulfamethoxazole for Strains of Bordetella pertussis isolated in the Czech Republic in 1967–2015. Cent Eur J Public Health. 2017;25(4):282–6. 29346850 10.21101/cejph.a4948

[26] Li L, Deng J, Ma X, et al. High Prevalence of Macrolide-Resistant Bordetella pertussis and ptxP1 Genotype, Mainland China, 2014–2016. Emerg Infect Dis. 2019;25(12):2205–14. 31742507 10.3201/eid2512.181836 PMC6874251

[27] Li Y, Liu X, Zhang B, He Q, Wang Z. Where macrolide resistance is prevalent. APMIS. 2015;123(4):361–3. 25703275 10.1111/apm.12357

[28] Ohtsuka M, Kikuchi K, Shimizu K, et al. Emergence of quinolone-resistant Bordetella pertussis in Japan. Antimicrob Agents Chemother. 2009;53(7):3147–9. 19414571 10.1128/AAC.00023-09 PMC2704657

